# Obstetric complications and socio-demographic characteristics associated with severe maternal morbidity at Mbeya Zonal Referral Hospital, Tanzania: a case–control study

**DOI:** 10.3389/fgwh.2026.1709603

**Published:** 2026-04-10

**Authors:** Erick Justin Mbogoro, Mathew Senga, Rebecca Mokeha

**Affiliations:** 1 Department of Mathematics and Statistics, University of Dodoma, Dodoma, Tanzania; 2 Department of Sociology and Anthropology & Population Studies and Research Centre, University of Dar es Salaam, Dar es Salaam, Tanzania; 3 Department of Reproductive and Child Health, Mbeya Zonal Referral Hospital, Mbeya, Tanzania

**Keywords:** case-control study, obstetric complications, severe maternal morbidity, socio-demographics, Tanzania

## Abstract

**Introduction:**

The issue of severe maternal morbidity continues to pose a significant public health challenge globally and has recently emerged as a complementary metric for evaluating the quality of maternal health care. Despite reductions in the maternal mortality ratio in Tanzania, severe maternal morbidity remains a significant threat to women of reproductive age. The aim of this study was to examine obstetric complications and the socio-demographic characteristics that are associated with severe maternal morbidity among women who utilized maternal health services at the Mbeya Zone Referral Hospital in Tanzania.

**Methods:**

A facility-based unmatched case-control study was conducted on 768 women (256 cases and 512 controls) at the Mbeya Zonal Referral Hospital in Tanzania. All inpatients fulfilling the criteria of severe maternal morbidity and non-severe maternal morbidity during the study period were eligible for this study. For each identified case, two corresponding women were randomly selected as controls. Bivariate logistic regression analyses were conducted to identify variables for inclusion in the multivariable logistic regression model.

**Results:**

The hospital-based severe maternal morbidity incidence ratio was 75.4 per 1,000 live births. Hypertensive and haemorrhagic disorders were the most morbid conditions, comprising 139 (54.3%) and 88 (34.4%), respectively. Women aged 35 years or older (AOR = 2.248, 95% CI: 1.446–3.494), living without a partner (AOR = 2.313, 95% CI: 1.355–3.948) and residing in rural areas (AOR = 2.503, 95% CI: 1.783–3.514) were found to significantly increase the risk of severe maternal morbidity. However, younger women (AOR = 0.480, 95% CI: 0.256–0.890) and those with post-secondary education (AOR = 0.497, 95% CI: 0.297–0.833) revealed a protective effect against severe maternal morbidity.

**Conclusion and recommendations:**

Severe maternal morbidity remains a challenging problem in the Southern Highlands of Tanzania. The findings highlight sociodemographic factors, such as age, marital status, education levels, and place of residence, as significant predictors of severe maternal morbidity. Therefore, integrated strategies targeting specific obstetric complications and relevant sociodemographic factors are essential for effective mitigations.

## Introduction

Pregnancy, childbirth, or the postpartum period are the times of greatest lifetime risk of adverse maternal outcomes (1). Maternal health outcomes can be conceptualised on a continuum of severity, which begins with healthy pregnant women, followed by non-severe morbidity, severe morbidity, and ultimately deaths (2). The Maternal Morbidity Working Group (MMWG) defines maternal morbidity as any health condition contributed by or complicating pregnancy and childbirth that adversely affects a woman's well-being or functioning (3). The World Health Organisation (WHO) defines Severe maternal morbidity (SMM) as “potential life-threatening condition that occurs during pregnancy, childbirth or during the postpartum period from which maternal near-miss events would emerge (4). This includes a set of heterogeneous maternal conditions known to be associated with severe illness, prolonged hospitalisation, or high case-fatality (5). Severe maternal morbidity, including life-threatening conditions, has emerged as a supplementary indicator for measuring the quality of maternal health care (3, 6).

Severe maternal complications, are the major global public health issues which impose socioeconomic burdens on individuals, families, societies, and the health system (7). The global efforts to combat maternal morbidity have been adopted by the new Sustainable Development Goals (SDG 3) agenda, with the target of ending preventable maternal mortality (EPMM) by addressing the overall health needs of girls and women (8). The agreement is to reduce the global maternal mortality ratio to 70 per 100,000 live births, with no country exceeding two times that ratio by 2030 (8). Despite the efforts of SDG 3, literature has indicated that severe maternal morbidity is several times more common than mortality among pregnant women (3, 9). It is estimated that 20 to 30 women suffer from acute maternal morbidity for every maternal death (3). Globally, about 80 million cases of acute maternal disorders were estimated in 2017 (10). Sub-Saharan Africa (SSA) is the region in the world that bears the greatest global burden of SMM (11). An estimate of SMM of 198 per 1,000 live births has been reported in SSA, and the burden was highly contributed by haemorrhage and hypertensive disorders (12).

Recent data indicate a notable reduction in the pregnancy-related mortality ratio in Tanzania, which has decreased from 556 per 100,000 live births in 2016 to 104 per 100,000 live births, with a confidence interval of 59 to 149 per 100,000 live births in 2022 (13). While this decline in maternal mortality is encouraging, there remains a significant gap in knowledge regarding the burden of SMM cases that threatens the survival and long-term effects of women of reproductive age in Tanzania. For instance, it is estimated that around 3,000 women in Tanzania are diagnosed with obstetric fistulas annually, suggesting that many more women endure this debilitating condition (14). This condition exemplifies a severe maternal complication arising from childbirth injuries, which is linked to various forms of physical, socioeconomic, and psychological distress (15). Detailed review of medical records is currently considered the optimal way to identify the true cases of SMM (16). However, the prevalence of SMM within the country remains unknown, reflecting the gaps in systematic review practices and maternal health surveillance systems.

Maternal sociodemographic characteristics play a crucial role in shaping maternal health outcomes. Among these factors, maternal age at childbearing is an important determinant of pregnancy outcomes (13). Scholars associate teenage pregnancy (pregnancy before the age of 20) with adverse maternal outcomes, as biological and physical immaturity can limit the body's ability to handle a healthy pregnancy (17). Also, inadequate pelvic development increases the risk of restricted expansion during childbirth, often resulting in obstructed labour. Elderly pregnant women are at higher risk of comorbidities such as hypertension, diabetes mellitus, and obesity (18). They also face an increased likelihood of recurrent pregnancy complications from previous pregnancies (18). Extensive research has shown that wealth, education, marital status, place of residence and health insurance are crucial factors influencing women's access to maternal healthcare and the utilisation of skilled health attendants (19, 20, 22); eventually, these would have an influence on the maternal health outcomes. However, these factors are more likely to limit women's ability to protect their health.

Earlier studies in different settings examined the association between SMM and some of the sociodemographic characteristics such as age (21–23), marital status (9, 22, 23), education levels (24–27), occupations (23), place of residence (24, 28) and medical insurance (23). It has been observed that some of the studies applied diverse criteria or definitions for identifying cases of SMM, which emphasises the need for further studies in different settings. However, there are limited studies in Tanzania that have explored the associations between sociodemographic characteristics and SMM based on the medical records. Evaluating the odds of SMM, focusing on the women's socio-demographic characteristics as determinants, may enhance better identification and understanding of those at higher risk among women of reproductive age.

Identifying the obstetric complications and determinants of SMM is crucial for strengthening the healthcare system to avoid similar cases in the future and subsequently reducing maternal mortality (28). Therefore, the aim of this study was to examine obstetric complications and the socio-demographic characteristics that are associated with severe maternal morbidity among women who utilized maternal health services at the Mbeya Zone Referral Hospital in Tanzania. It was anticipated that these estimations would provide valuable insights for maternal health professionals and policymakers, thereby facilitating a more effective and tailored approach to addressing the unique needs of women at risk of severe maternal morbidity.

## Methods and materials

### Study design and area

This was a facility-based unmatched case-control study, with data extraction occurring from January to August 2023 at the Mbeya Zonal Referral Hospital (MZRH). Recognised as the oldest and largest public hospital in the southwestern regions of Tanzania's Southern Highlands, it serves as a teaching hospital affiliated with the University of Dar es Salaam, Mbeya College of Health and Allied Sciences. Furthermore, it serves as a zonal tertiary referral hospital, providing medical services to an estimated 10 million people across the Tanzania Southern Highlands Regions (29). The hospital is made up of three campuses: the Main Hospital, the Maternity Wing (META), which is a study site, and the New Paediatric Wing. The Maternity Wing of MZRH offers comprehensive obstetrics care, including services for low-risk pregnancies, specialized clinics for high-risk expectant mothers, and advanced obstetric care for patients referred from lower-tier health facilities including 7 tertiary regional hospitals across the Tanzania Southern Highlands zone. The maternity wing is equipped with 206 beds, accommodating antenatal, labour, and postnatal wards. Additionally, the wing features with blood bank unit, an Intensive Care Unit (ICU) that operates under continuous medical supervision and provides mechanical ventilation for patients requiring intensive care. Annually, the hospital records approximately 5,351 deliveries, with approximately 450 deliveries per month. The institution is well-regarded for its training and research capabilities, largely due to the presence of the Mbeya Medical Research Centre (MMRC). Furthermore, it boasts advanced medical infrastructure and a team of skilled gynaecologists adept at diagnosing and interpreting maternal adverse outcomes effectively.

### Population

The study population comprised all women of reproductive age who sought maternal health services at the MZRH during the pregnancy, childbirth, or within the first 42 days of postpartum. According to the 2022 Tanzania Population and Housing Census (30), the Southern Highlands Regions are predominantly rural, with approximately 72.1% of residents living in these areas. The majority of the people relies on agriculture, forestry, and fishing as their primary sources of income and food security. The average age at first marriage for females aged 15 and older in this zone is 21.8 years. Additionally, the adult literacy rate for persons aged 15 and above is approximately 82.8%, although it remains higher among males than females. The census also highlights a growing trend of female-headed households owning houses across the regions in the Southern Highlands.

### Sample size and sampling procedure

The sample size was determined utilizing the Epi Info 7.2.4.0 software, specifically the StatCalc menu designed for Unmatched Case-Control studies. The Kelsey sample size methodology was selected for this investigation. The parameters employed in the sample size calculation included a power of 80% with a 95% Confidence Interval (CI), a case-to-control ratio of 1:2 (two controls for each case), a hypothetical odds ratio of 2, and a prevalence of exposure of 13.6% among cases, contrasted with 7.2% among controls, specifically for those with a parity of five or more, as referenced from a study conducted in a neighbouring country (23). Consequently, the minimum sample size was calculated to be 768 women, consisting of 256 cases and 512 controls.

All inpatients fulfilling the criteria of severe maternal morbidity (cases) and non-severe maternal morbidity (controls) were eligible for this study. The cases and controls were identified using the maternal conditions and clinical procedures outlined in the Standard Treatment Guidelines (STG) for Tanzania Mainland (31), which adapts World Health Organisation (WHO) criteria with minor modifications to identify cases for low-resource settings (e.g., the threshold for blood transfusion). These criteria were further supplemented by evidence from published literature from other settings (5, 32, 33) to ensure contextual relevance. All inpatients who survived severe maternal morbidity were included based on the presence of at least one clinical condition confirmed by clinical diagnosis, laboratory results, treatment, or medical interventions. Consecutive sampling was employed for the selection of cases until the sample size was achieved. Women who received maternal health services during the same period as the cases and were clinically diagnosed with obstetric morbidities that did not meet the case criteria were considered eligible as controls. This approach ensured consistency in medical records and diagnosis practices. The controls were selected using a simple random sampling procedure, considering the ratio of two controls per case (2:1). A lottery-based method using patients' identification numbers was employed to randomly select controls from those clinically identified with non-severe maternal morbidity. The selection of the controls was based on at least one of the characteristics similar to the cases, such as maternal age, mode of delivery, gestation age, parity, or gravida. The study excluded women with non-obstetric conditions unrelated to pregnancy, gynaecological issues (e.g., ovarian cysts, fibroids, cancers, etc.) and those with incomplete medical records that hindered accurate classifications of the outcomes and statistical analyses. The list of maternal conditions and criteria for the review, are presented in additional file 1 (Supplementary Table S1).

### Data collection and procedure

The established list of maternal conditions with clinical parameters that distinguish between severe maternal morbidity and without severe maternal morbidity was made available to research assistants (midwives), and gynaecologists participated in the study. Furthermore, the data collection instrument designed for this research was made accessible in the maternity department of the hospital and was systematically completed by research assistants, once the inpatients satisfied the inclusion criteria. The four research assistants meticulously examined the inpatient registers and various medical records across antenatal wards, delivery rooms, postnatal wards, and the intensive care unit (ICU). Relevant information pertaining to both women with severe and without severe maternal morbidity was consistently extracted following discharge. The extracted information includes sociodemographic characteristics, obstetric complications, and intervention strategies. The information documented by the research assistants underwent a verification process conducted by gynaecologists prior to the initiation of data entry. The gynaecologists overseeing the study played a crucial role and participated in training alongside the midwives recruited from the maternity department. Additionally, data regarding the total number of women who accessed maternal health services and the count of live deliveries and births during the study period were sourced from the hospital database and birth registers.

### Data quality control and assurance

The data collection instrument designed for this study was prepared and reviewed by the Mbeya Medical Research and Ethical Review Committee as part of the protocol review. To ensure the accuracy and consistency of the data abstraction tool, a pre-test was conducted at a regional referral hospital not included in the study. This allowed assessment of the appropriateness of the tool and identification of necessary adjustments before the actual study began. Four midwives at the MZRH were hired and comprehensively trained for data abstraction. Additionally, the gynaecologist in charge of this study reviewed the extracted data to verify its accuracy before entering it in the computer software for further analysis.

### Variables of the study

The outcome variable is severe maternal morbidity. Patients were assigned a code of 1 if they experienced severe maternal morbidity and a code of 0 if they did not. The independent variables in this analysis were obtained from the maternal medical records. The sociodemographic characteristics assessed in this study were maternal age, marital status, education levels, occupation status, medical insurance, place of residence, and parity. The variables were categorised as follows: maternal age in years (less than 20, 20–29, 30–34, and 35 or older), marital status (not living together and living together), education level (no formal or primary, secondary and post-secondary), occupation (employed/professional, not employed, peasants, and business), medical insurance (no and yes), place of residence (rural and urban), and parity (0, 1, 2 to 4, 5 or more). These variables were included in the analysis to assess their effects on the outcome variable.

### Data processing and analysis

Data were systematically entered, cleaned, and analysed utilizing IBM SPSS Statistics version 27. A descriptive analysis was conducted, summarizing categorical variables in terms of frequencies and percentages. Continuous variable was represented by their mean and standard deviation. The associated factors between two groups (SMM and without SMM) were initially evaluated using the Chi-square tests. Additionally, bivariate and multivariable logistic regression analyses were executed to identify the socio-demographic factors associated with severe maternal morbidity, thereby estimating both unadjusted and adjusted odds ratios, along with their corresponding 95% Confidence Intervals (CI). All variables examined in the descriptive analysis were included in the bivariate logistic regression analysis to evaluate the crude effect of each exposure variable and its categories to the outcome of interest. Variables identified in the bivariate logistic regression analysis with a category *P*-value less than 0.25 were included in the multivariable logistic regression analysis. The model's fit was evaluated through the Hosmer and Lemeshow goodness-of-fit tests and Nagelkerke R² to assess explanatory power. A backward stepwise approach was adopted for variable selection, iterating the process until the statistically significant variables were identified and included in the final model. Model diagnostics indicated adequate fit (Hosmer-Lemeshow $x^{2}=5.557$, *P* = 0.592; Nagelkerke *R*^2^ = 0.123). A summary of the model diagnostics is provided in Supplementary Table S2.

## Results

**Figure 1:**
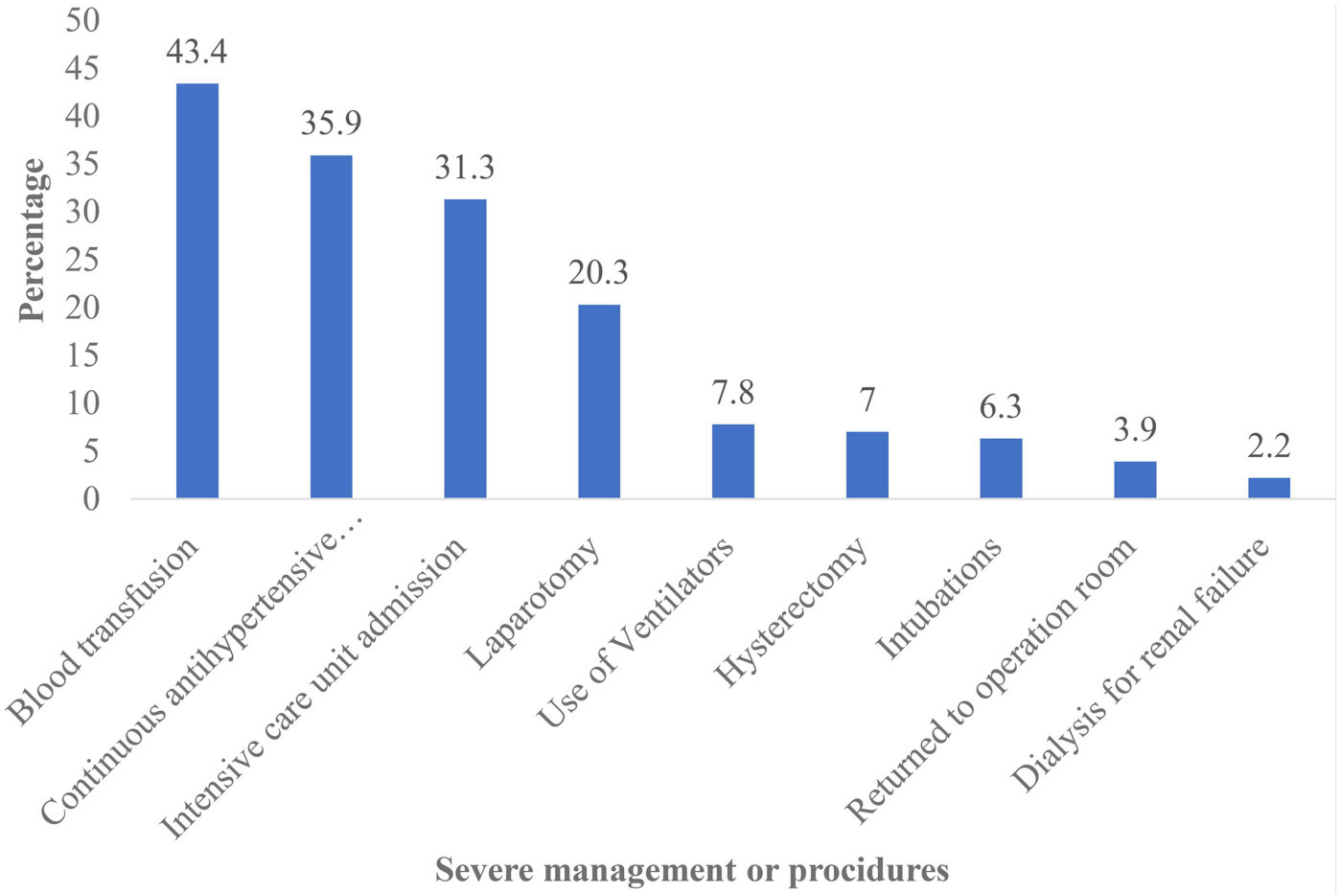
Proportions of severe maternal morbidity procidures at MZRH (*n* = 256).

A total of 5,311 women inpatients and 20,106 outpatients accessed maternal health services at the MZRH, resulting in an aggregate of 25,417 patients. Throughout this timeframe, the MZRH recorded a total of 3,477 deliveries and 3,397 live births. From this population, 256 women experienced severe maternal morbidity (cases) and 512 women without such morbidity were designated as controls, culminating in a total sample size of 768. For the entire study period, the facility-based severe maternal morbidity incidence ratio was 75.4 per 1,000 live births. Among women who survived severe maternal morbidity, hypertensive and haemorrhagic disorders were the most morbid conditions, comprising 139 (54.3%) and 88 (34.4%), respectively (Table 1). Furthermore, blood transfusion was identified as the most severe management indicator, accounting for 111 (43.4%) of the cases, followed by continuous antihypertensive medications, admissions to the intensive care unit and laparotomy, which constitute 92 (35.9%), 80 (31.3%) and 52 (20.3%), respectively (Figure 1).

**Table 1:** Underlying obstetric complications among women with severe (cases) and without severe (controls) maternal morbidity at the MZRH, Tanzania.

Morbidity conditions	Cases (*n* = 256)	Controls (*n* = 512)
	*n* (%)	*n* (%)
Haemorrhagic disorder	88 (34.4)	220 (43.0)
Postpartum haemorrhage (PPH)	58 (22.7)	173 (33.8)
Ectopic pregnancy	20 (7.8)	5 (1.0)
Uterine rupture	7 (2.7)	3 (0.6)
Abruption placentae	6 (2.3)	15 (2.9)
Placenta accreta/increta/percreta	2 (0.8)	2 (0.4)
Placenta previa	2 (0.8)	22 (4.3)
Hypertensive disorders	139 (54.3)	68 (13.3)
Hypertension encephalopathy	0 (0)	2 (0.4)
Pre-eclampsia	86 (33.6)	37 (7.2)
Eclampsia	42 (16.4)	0 (0)
HELLP syndrome	7 (2.7)	0 (0)
Puerperal sepsis	18 (7.0)	5 (1.0)
Endometritis	4 (1.6)	0 (0)
Pulmonary oedema	13 (5.1)	0 (0)
Pulmonary embolism	5 (2.0)	1 (0.2)
Respiratory failure	16 (6.3)	0 (0)
Thyroid crisis	2 (0.8)	0 (0)
PROM/PPROM	3 (1.2)	62 (12.1)
Cardiac condition	4 (1.6)	0 (0)
Malaria in pregnancy	8 (3.1)	7 (1.4)
Gestational anaemia	42 (16.4)	22 (4.3)
Peripartum cardiomyopathy (PPCM)	11 (4.3)	0 (0)
Gestational diabetes mellitus	9 (3.5)	7 (1.4)
Obstructed labour	11 (4.3)	109 (21.3)
Renal failure	6 (2.3)	0 (0)
Psychiatric disorder	2 (0.8)	6 (1.2)
Pregnancy loss/abortion	8 (3.1)	58 (11.3)

HELLP, Homolysis, elevated liver enzymes, and low platelet; PROM/PPROM, Preterm premature rupture of membranes.

### Sociodemographic characteristics

Table 2 shows that the mean age of the cases [29.5 (SD ± 6.8) years] was higher than that of the controls [27.6 (SD ± 6.1) years]. A higher proportion of women aged 35 an above appeared among the cases than the controls (27.3% vs. 13.5%). Additionally, 8.2% of the cases were younger than 20 years, compared to 11.5% among the controls. Women who lived without partners, had primary or no formal education, and those with peasant occupations constituted higher proportions of the cases than the controls (14.5% vs. 10.2%, 48.4% vs. 33.6%, and 40.2% vs. 26.4%, respectively). A higher proportion of women residing in rural areas and those with para five or more were observed among the cases (43.4% vs. 22.3% and 12.1% vs. 4.3%, respectively). The proportion of women with medical insurance was smaller among the cases than the controls (15.2% vs. 20.9%). As shown in Table 1, the comparison between cases and controls revealed statistically significant differences (P<0.05) in age, education levels, occupations, place of residence, and parity. However, the comparison between two groups revealed statistically significant differences (P<0.1) in marital status and medical insurance.

**Table 2:** Sociodemographic characteristics of the women with severe (cases) and without severe (controls) maternal morbidity at the MZRH, Tanzania.

Variables	Cases (*n* = 256)	Controls (*n* = 512)	Total (*n* = 768)	*P*-value
	*n* (%)	*n* (%)	*n* (%)	
Age in years	29.5^a^ ± 6.8^b^	27.6^a^ ± 6.1^b^		
Age groups				<0.001**
<20	21 (8.2)	59 (11.5)	80 (10.4)	
20–29	102 (39.8)	248 (48.4)	350 (45.6)	
30–34	63 (24.6)	136 (26.6)	199 (25.9)	
>=35	70 (27.3)	69 (13.5)	139 (18.1)	
Marital status				0.079*
Not living together	37 (14.5)	52 (10.2)	89 (11.6)	
Living together	219 (85.5)	460 (89.8)	679 (88.4)	
Education levels				<0.001**
Non formal or Primary	124 (48.4)	172 (33.6)	296 (38.5)	
Secondary	105 (41.0)	254 (49.6)	359 (46.7)	
Post-secondary	27 (10.5)	86 (16.8)	113 (14.7)	
Occupations				<0.001**
Employed/Professional	37 (14.5)	93 (18.2)	130 (16.9)	
Not employed	51 (19.9)	152 (29.7)	203 (26.4)	
Peasants	103 (40.2)	135 (26.4)	238 (31.0)	
Business	65 (25.4)	132 (25.8)	197 (25.7)	
Place of residence				<0.001**
Rural	111 (43.4)	114 (22.3)	225 (29.3)	
Urban	145 (56.6)	398 (77.7)	543 (70.7)	
Medical insurance				0.059*
No	217 (84.8)	405 (79.1)	622 (81.0)	
Yes	39 (15.2)	107 (20.9)	146 (19.0)	
Parity				<0.001**
0	21 (8.2)	70 (13.7)	91 (11.8)	
1	80 (31.3)	209 (40.8)	289 (37.6)	
2–4	124 (48.4)	211 (41.2)	335 (43.6)	
5+	31 (12.1)	22 (4.3)	53 (6.9)	

^a^ Mean.

^b^ Standard deviation.

* at p<0.1; $\chi^{2}$ is Chi-square.

** Statistically significant at p<0.05.

### The results of the multivariable logistic regression model

The results of the adjusted logistic regression model (Table 3), show that age of the mother, marital status, education levels, and place of residence were significant determinants of severe maternal morbidity. Women aged 35 years or older had 2.248 times more likely of experiencing severe maternal morbidity compared to women aged 20 to 29 years (AOR = 2.248, 95% CI: 1.446–3.494). However, women of age under 20 years were associated with reduced odds of severe maternal morbidity by 52% (AOR = 0.480, 95% CI: 0.256–0.900) than those aged 20 to 29 years. Women living without partners were 2.313 times more likely of experiencing severe maternal morbidity (AOR = 2.313, 95% CI: 1.355–3.948) compared to their counterparts. Nevertheless, women with post-secondary education were about 50% less likely of experiencing severe maternal morbidity (AOR = 0.497, 95% CI: 0.297–0.833) compared to women with primary or no formal education. Further, women dwelling in the rural areas were 2.503 times more likely of experiencing severe maternal morbidity (AOR = 2.503, 95% CI: 1.783–3.514) compared to their counterparts.

**Table 3:** Bivariate and multivariable logistic regression models for the sociodemographic characteristics associated with severe maternal morbidity at the MZRH, Tanzania.

Variables	Unadjusted OR (95%CI)	*P*-value	Adjusted OR (95%CI)^a^	*P*-value
Maternal age		<0.001		<0.001
<20	0.865 (0.500–1.498)	0.606	0.480 (0.256–0.900)	0.022
20–29	Ref.		Ref.	
30–34	1.126 (0.772–1.643)	0.537	1.201 (0.809–1.782)	0.364
>=35	2.467 (1.646–3.697)	<0.001	2.248 (1.446–3.494)	<0.001
Marital status				
Living together	Ref.		Ref.	
Not living together	1.495 (0.952–2.347)		2.313 (1.155–3.948)	0.002
Education levels		<0.001		0.028
Primary or no formal	Ref.		Ref.	
Secondary	0.573 (0.415–0.793)	< 0.001	0.783 (0.547–1.122)	0.183
Post-secondary	0.435 (0.267–0.711)	< 0.001	0.497 (0.297–0.833)	0.008
Occupations		<0.001		
Employed/Professional	0.521 (0.329–0.825)	0.005	–	–
Not employed	0.440 (0.292–0.661)	<0.001	–	–
Peasants	Ref.		–	–
Business	0.645 (0.436–0.956)	0.029	–	–
Place of residence				
Rural	2.673 (1.935–3.692)	<0.001	2.503 (1.783–3.514)	<0.001
Urban	Ref.		Ref.	–
Medical insurance				
No	Ref.		–	–
Yes	0.680 (0.455–1.017)	0.060	–	–
Parity		<0.001		
0	0.510 (0.299–0.872)	0.014	–	–
1	0.651 (0.464–0.915)	0.013	–	–
2–4	Ref.		–	–
5+	2.398 (1.330–4.324)	0.004	–	–

^a^ Adjusted for variables included in the final model; OR, Odds Ratio; CI, Confidence interval; Ref, Reference category is 1.000; –, Indicates variable excluded from the final model due to confounding effect.

## Discussion

The objective of this study was to examine the sociodemographic characteristics associated with severe maternal morbidity among women received maternal health services at the MZRH. Our study revealed a severe maternal morbidity ratio of 75.4 per 1,000 live births which is slightly higher than the facility-based case ratio of 73.8 per 1,000 live births found in Southern Ethiopia (34). The high burden observed in this study is likely attributable to the hospital's role as the sole tertiary referral facility for the Tanzania Southern Highlands, serving a population of approximately 10 million and managing all complex obstetric emergencies in this zone. Among women with severe maternal morbidity (SMM) hypertensive and haemorrhagic disorders were the most morbid conditions diagnosed during the study period at the MZRH. This findings concurred with a prospective case control study conducted in Rwandan district hospitals (23). Furthermore, blood transfusion was identified as the most severe management indicator, followed by antihypertensive medications and admissions to the intensive care unit. Identification of morbid conditions and severe management indicators based on hospital maternal records review presents opportunities to enhance care, reduce preventable cases of SMM, and avert maternal mortality (35). Moreover, the study found that maternal age, marital status, education level, and place of residence are significant predictors of severe maternal morbidity.

Maternal age remains a vital determinant of obstetric outcomes in different settings. According to the multivariable logistic regression analysis, the likelihood of severe maternal morbidity was two times higher among women aged 35 years or older than among those aged 20 to 29 years. This finding is consistent with the results found in the case-control studies in the other settings (22, 23). It has been indicated in the literature that older women are more likely to have underlying comorbid conditions such as obesity, diabetes, recurrent maternal complications and hypertensions (36, 37). This finding aligns with national evidence from the 2022 Tanzania Demographic and Health Survey (TDHS), which shows that the age-specific maternal mortality rate is highest among women aged 35–39 and lowest among women aged 25–29 (13). Thus, strengthening age-specific obstetric care and interventions would provide the opportunity to reduce the challenges faced by older pregnant women and ultimately avert maternal deaths.

The findings of the present study unexpectedly revealed that women under 20 years of age were significantly less likely to experience severe maternal morbidity. This result contrasts with the research conducted by (38), and (23) in Brazil and Rwanda, which demonstrated that adolescent mothers faced increased odds of SMM when analysed through a multivariable logistic regression model. The result is also contrary to the earlier study by (21), which found that the odds of SMM were higher in mothers at both ends of the age spectrum, such as those aged 35 years or older and adolescent mothers. Still, some evidence from the literature suggests that teen mothers are at risk of eclampsia and obstructed labour (39), unsafe abortions, haemorrhage and sepsis (40). Indeed, the protective effect of younger maternal age against SMM in this study should be interpreted with caution. This study relied on medical records available at the health facility, which means the findings are dependent on the documentation and the characteristics of the population served. However, in our sample, only 10.4% of women were under 20 years of age, which may have reduced the statistical power to detect risks in this subgroup. The under-representation of teen mothers is aligned with national data from the 2022 TDHS, which found that 22% of women under 20 had ever been pregnant and 16% had experienced a live birth (14).

Besides the maternal age, the study assessed the influence of marital status on severe maternal morbidity. Findings indicated that non-partnered women were 2.3 times more likely to experience SMM than their counterparts. The result was similar to previous case-control studies elsewhere (9, 22, 23), which also found that non-partnered women had higher odds of SMM. It has been shown in the literature that non-partnered women had a higher likelihood of depressive and anxiety disorders as well as experiencing self-harm than partnered pregnant women (41). This aligned with a meta-analysis done by (42) that revealed that unmarried women were five times more likely to have antenatal depression compared to married women. In addition, literature demonstrated that non-partnered women had a higher chance of inadequate prenatal care services due to little or no motivation to use the services, a lack of social, financial, and affective support, as well as incentives to care for themselves, which eventually exposed them to various maternal complications (42, 43). This finding is aligned with evidence showing higher odds of depression among unmarried pregnant women in Tanzania (44), a condition that may increase vulnerability to severe maternal complications. It is worth noting that couple support during pregnancy, childbirth, or the postpartum period is crucial for the well-being of a mother and child. Such findings call for the government and other stakeholders to encourage partners' involvement in maternal health services.

Women education attainment remains as a crucial social determinant of maternal health outcomes in different settings. The results show that education was a significant predictor of severe maternal morbidity. In particular, this study showed that post-secondary education was found to be protective, reducing the likelihood of severe maternal morbidity by 51% relative to women with primary or no education. The result concur with other studies elsewhere, which found that the odds of severe maternal morbidity decreased with an increase in mothers education levels (24–27). In contrast, a multicentre prospective case-control study in Rwanda through district hospitals found no association between education and severe maternal morbidity (23). Indeed, women with higher levels of education are significantly more likely to make optimal use of maternal health care services (45) and to be aware of the importance of seeking quality health services (26), which ultimately reduces the risk of severe complications. This aligns with national evidence from the 2022 TDHS, which shows that women with secondary or higher education are significantly more likely to utilise maternal health services compared to those with little or no education (13). This finding underscores the importance of women's education and maternal health education programs in reducing the risk of severe complications and enhancing their ability to make informed decisions regarding their reproductive health.

Our study highlights significant geographic disparities, revealing a robust correlation between maternal residence and the likelihood of experiencing severe maternal morbidity. Notably, women residing in rural areas face a 2.5-fold increased likelihood of experiencing severe maternal morbidity compared to their counterparts. This finding aligns with previous studies conducted by (24) in Ethiopia and (28) in Mogadishu, Somalia, both of which established that rural residency had increased risks of severe maternal morbidity. The disparity in the odds of experiencing severe maternal morbidity (SMM) between rural and urban populations in Tanzania reflects inequalities in maternal healthcare access and quality, suboptimal utilisation of antenatal care (ANC), and low uptake of timely postnatal care (PNC) among rural women (14), thereby increasing the likelihood of severe complications. Consequently, the persistent challenge of SMM among rural populations underscores the need for targeted public health interventions like strengthening the health system, referral and transport systems, enhancing maternal healthcare practices, and engaging community health workers, especially in settings with limited resources and inadequate obstetric care services.

### Strengths and limitations of the study

This study represents one of the few exceptional studies conducted at a zonal tertiary referral hospital in Tanzania, focusing on women with severe maternal morbidity (cases) and without such morbidity (controls). Nonetheless, it is important to acknowledge certain limitations inherent in this study. The data were sourced from a zonal tertiary referral hospital, which may restrict the applicability of the findings to other contexts or populations. The study relied on administrative records, the quality of which depends on the accuracy of the recorded information. The records may have inconsistencies that may have led to misclassification of severity, and this should be considered when interpreting the findings. To mitigate this issue, we implemented a quality review of the data abstraction tool and the collected data before further analysis. The identification of cases of severe maternal morbidity was based on the Standard Treatment Guideline for Mainland Tanzania. This guideline adapts the WHO criteria for severe maternal morbidity, with some modifications for low-resource settings. Also, these should be considered when interpreting the findings of the current study.

## Conclusion

The present study shows that, although the hospital-based severe maternal morbidity (SMM) ratio is lower than the highest estimate ever reported in Sub-Saharan Africa (SSA), adverse maternal conditions remain a significant challenge in the Southern Highlands of Tanzania. The multivariable logistic regression model yielded significant insights into the relationship between severe maternal morbidity and various sociodemographic factors, such as maternal age, marital status, education levels, and place of residence. The risk of SMM was higher among women aged 35 years and above, women not living with partners, and those living in rural areas. Moreover, there is a protective effect against SMM for women with post-secondary education. The findings of this study underscore the need for tailored maternal health care services and targeted interventions that address the specific reproductive health needs of at-risk populations. Therefore, implementing integrated strategies that address preventable obstetric causes and relevant sociodemographic factors is essential for effectively mitigating risks and delivering maternal services to the populations most in need. These insights can guide the development or refinement of policies and protocols to better maternal health outcomes in Tanzania.

## Data Availability

The datasets presented in this article are not readily available because the study extracted data from the medical records of patients, which are restricted from public access in accordance with Tanzanian regulations governing the utilization and dissemination of health data. Nevertheless, the data set can be obtained from the corresponding author upon reasonable request with permission from the Ministry of Health in Tanzania. Requests to access the datasets should be directed to Mbeya Zonal Referral Hospital, https://www.mzrh.go.tz.
