# Supplementary material for: Obstetric complications and socio-demographic characteristics associated with severe maternal morbidity at Mbeya Zonal Referral Hospital, Tanzania: a case–control study
Source: Front Glob Womens Health. 2026 Apr 10;7:1709603. doi: 10.3389/fgwh.2026.1709603 (PMC13106486; doi:10.3389/fgwh.2026.1709603)
Supplement: Supplementary file 1 [file Table1.docx]

| **Table S1: List of severe and not severe maternal morbidity, clinical diagnosis and management or procedures.** | | |
| --- | --- | --- |
| **Maternal conditions** | **Severe Maternal morbidity** | **Without severe morbidity** |
| **Obstetric haemorrhage**  Placenta previa  Placental abruption  Intrapatrum haemorrhage  Postpartum haemorrhage  Raptured uterus  Unplanned peripartum hysterectomy | Severe abdominal pain, Vaginal bleeding leading to hemodynamic instability fetal distress requiring emergency CS or interventions like unplanned hysterectomy, went to ICU for treatment, Blood transfusion of > 1 unit. | With no emergency CS, no hysterectomy, at most one unit of blood transfusion, went to ICU for observations only, planned peripartum hysterectomy. |
| Obstructed labour | Prolonged labour,  severe moulding + 3,  fetal distress,  emergency CS,  instrumental delivery,  uterine rupture (before or during labour) and  Fistula. | Early obstructed labour without fetal distress, uterine rupture or fistula formation. |
| Anaemia | Haemoglobin <7g/dl, anaemia with heart failure, and anaemia warranting blood transfusion (at least 2 units) in third trimester. | Anaemia in the 1^st^ and 2^nd^ trimester, anaemic between 7 g/dl -9g/dl in the 3^rd^ trimester. |
| **Hypertension disorder**  Pre-eclampsia | When BP ≥ 140/90 after 20 weeks GA and proteinuria +2 or more, BP≥140/90 with features of end organ dysfunction (Renal, liver failure, pulmonary adema, loss of vision), stroke and coma. | Blood pressure control with little oral medications or hypertension that does  Not require intravenous medications. |
| Eclampsia | BP > 160/110mm Hg with proteinuria, loss of consciousness, chronic seizures, coma, loss of vision and continuous infusion of an antihypertensive medication. |  |
| HELLP ((Haemolysis, Elevated Liver enzymes, Low Platelet count) syndrome. | Severe haemolysis, platelets < 50,000/mm3; elevated liver functions (serum glutamic oxaloacetic transaminase > 70 U/L, low platelets count needing platelet transfusion, ALT<70, transaminase, severe liver injury admitted to ICU. | Abnormal liver function requiring extra prolonged postpartum length of stay but not in the ICU, platelets between (50,000-1000000)/mm3, doesn’t require any transfusion |
| Pulmonary embolism | Acute onset of shortness of breath (dyspnea), diminished oxygen saturation (oxygen saturation <90% for >60 min), edema, diminished breath sounds, pleuritic chest pain, tachypnea and obstetric shock. | Administration of oxygen Without established pulmonary diagnosis. |
| Renal complication | Treatment of acute renal failure with dialysis and oliguria treated with multiple doses. | Oliguria treated with only single dose of intravenous fluids and furosemide with no ICU admission. |
| Ectopic pregnancy | Ruptured ectopic pregnancy with blood transfusion (BT) depending on the blood loss, emergence laparotomy, use of antibiotic for 5-7 days. | Unruptured ectopic pregnancy treated medically with no BT, no emergence laparotomy. |
| Pregnancy loss/abortions | Incomplete abortions needing BT, septic abortion, inevitable abortion with obstetric shock, coma, and BT if indicated. | Incomplete abortion without BT, threatened abortion and inevitable abortion. |
| Cardiac disease  and peripartum cardiomyopathy | Congenital heart disease with ICU admission and require other significant interventions or treatments | Congenital heart disease with ICU admission for observation only or without ICU admission. |
| Sepsis or systemic infections | High fever $>{38}^{o}C$ with septic shock, PROM chorioamnionitis, pulmonary complication, acute respiratory distress syndrome, foul smelling or purulent discharge managed with excessive antibiotics, and fluids, extended length of stay, emergence hysterectomy | Positive blood culture without other evidence of significant systemic illness. |
| Psychiatric disorder | Postpartum depression  schizophrenia | Postpartum blues  Anxiety |
| Diabetes mellitus (DM) | Uncontrolled DM  DM with complications like DKA and IUFD | DM controlled with medications |
| Prelabour Rupture of Membranes (PROM) and | PPROM with chorioamnionitis | PPROM, PROM without chorioamnionitis |
| Thyroid diseases | Thyroid crisis | Thyroid disease on treatment |
| Malaria in pregnancy | Complicated malaria | Non complicated malaria |
| Cervical tear/perineal tears | 3^rd^ and 4^th^ degree perineal tear, cervical tear needing BT | 1^st^ and 2^nd^ degree perineal tear |
| **Managements or procedures for severe maternal morbidity**  -Used blood products or had a blood transfusion $>$1units | | |
| -Extended intubation | | |
| -The use of a ventilator or mechanical ventilation  -Emergence or unplanned hysterectomy for any reason | | |
| -Return to operation room for major procedure | | |
| -ICU admission with treatment or diagnostic or therapeutic procedure | | |
| -Dialysis for renal failure | | |
| -Surgery or laparotomy excluding CS | | |
| -Continuous antihypertensive medications | | |
