# Supplementary material for: Obstetric complications and socio-demographic characteristics associated with severe maternal morbidity at Mbeya Zonal Referral Hospital, Tanzania: a case–control study
Source: Front Glob Womens Health. 2026 Apr 10;7:1709603. doi: 10.3389/fgwh.2026.1709603 (PMC13106486; doi:10.3389/fgwh.2026.1709603)
Supplement: Supplementary file 2 [file Table2.docx]

**Table S2: Modal Diagnostics Summary**

| Iteration | Variables retained | Variable removed | Hosmer-Lemeshow $x^{2}(p)$ | Nagelkerke$R^{2}$ |
| --- | --- | --- | --- | --- |
| Step 1 (Initial) | Age, marital status, education, occupations, place of residence, medical insurance and parity | - | 4.642 (0.795) | 0.14 |
| Step 2 | Age, marital status, education, place of residence, medical insurance and parity | Occupations | 12.332 (0.137) | 0.132 |
| Step 3 | Age, marital status, education, place of residence, parity | Medical insurance | 15.345 (0.053) | 0.131 |
| Step 4 (Final) | Age, marital status, education, place of residence | Parity | 5.557 (0.592) | 0.123 |
